# An ontological analysis of some biological ontologies

**DOI:** 10.3389/fgene.2012.00269

**Published:** 2012-11-26

**Authors:** Briti Deb

**Affiliations:** Bioinformatics Center, IIScBangalore, India

## Introduction

The functional importance of biological entities makes their understanding, analysis, and representation essential in modern biology. Arguably, semantic representation necessary for machine interoperability is a far more difficult task than syntactic representation, necessitating conceptual schema and ontologies for in-silico biological knowledge representation. Biological ontologies are increasingly being developed for prediction, big data integration in semantic web, visualization, unstructured data interpretation, annotation, and eHealth ontology. Despite being widely used, deficiencies exist (Kumar and Smith, 2003; Kumar et al., 2004; Mougin and Bodenreider, 2005; Pal, 2006; Schulz, 2006) in their concepts, relations, and frameworks in general, leading to difficulties in semantic interoperability and integration, and possibility of wrong prediction after using them. In this opinion article, I attempted for the first time (in my knowledge) to show that some characteristic inadequacies of biological ontologies could be detected and prevented by using the philosophically inspired OntoClean method (Guarino, 2002) and the top-level DOLCE ontology (Masolo et al., 2009), both of which have well-founded formal semantics, and finally proposed an outline of a novel ontology framework which aims to remove existing deficiencies. Though preliminary, my arguments suggest that it would be worthy to look deeper into the use of OntoClean and DOLCE toward detecting ontological inadequacies and improving them, a detailed analysis of which is left as a future work. I may state that, this discussion is not meant to criticize any of the ontologies, but to present some arguments on their respective design choices when seen in the light of OntoClean and DOLCE.

## Analysis with OntoClean and DOLCE

The OntoClean method proposes to tag concepts on a taxonomy according to the following philosophical meta-properties: rigid, anti-rigid, non-rigid, carry-identity-criterion, supply-identity-criterion, carry-unity, and carry-anti-unity. It must be noted here that, these assignments are not “definitive” (Guarino and Welty, 2004), rather it demonstrate logical consequences of making such choices. In the following, I present six cases and put forward my conjectures on detecting ontological inadequacies and solutions to correct them using the OntoClean method and DOLCE top-level ontology.

- OntoClean method suggests that, an entity has an essential property if that property is held by it all the time, and is rigid if all the instances possess that property (Guarino, 1998, 1999). Adult human beings would have an essential property of “adult behavior.” But due to the fact that Gene Ontology (GO) (Ashburner et al., 2000) terms are designed to be applied across many species, a term such as the “adult behavior” could lead to confusion when applied to unicellular organisms like amoeba. It could also be debated whether the GO term “adult behavior” is a rigid property or not, since all instances of human adults may not display adult behavior. I believe that modeling ontologies after considering essential and rigid properties of entity would prevent such an inadequacy.
- Identity criteria is used to recognize whether individual entities are the same or different (Guarino, 1998, 1999). Several characteristic inadequacies both in the GO and the Unified Medical Language System (UMLS) could be identified (Mougin and Bodenreider, 2005), as a result of the failure to draw distinction between continuant (i.e., endurant) and occurrent (i.e., perdurant) entities (Masolo et al., 2009), and between dependent (such as cellular motion, temperature, and mass) and independent entities (Kumar and Smith, 2003). In the UMLS, a function is a continuant which has a subsumption relation with a process (an occurant), which I believe could be a case of identity violation. Instead of using the subsumption (is_A) relation, using the “participate_In” relation such as, “A Continuant participate_In an Occurant” would bring in more ontological adequacy.
- The GO described the term “extracellular” as the space external to the outermost structure of a cell. A question could arise on deciding the location and/or the granularity level of the term extracellular (Kumar et al., 2004). This problem could be attributed to the fact that the GO has not explicitly modeled the identity criteria of entities such as the extracellular, to be able to recognize entities as the same or different entity, in addition to not recognizing the unity criterion necessary toward recognizing parts of these individual entities.
- According to the UMLS, an organism attributes is_A conceptual entity. Given the fact that, organism attribute is not necessarily dependent on mind (because all organisms need not have a mind), whereas a conceptual entity is necessarily dependent on mind, my conjecture is that identity criteria has been violated. Using the DOLCE top-level ontological distinctions, and reorganizing conceptual entity as an agentive-physical-object (DOLCE:APO) and organism attribute as a non-agentive-physical-object (DOLCE:NAPO) could have helped to detect such inconsistencies.
- In the GO, the term “GO:0020037:heme binding” is a molecular function. From (Guarino, 1999, 2002), I understand that material role is a role which is anti-rigid (−R), inherit identity (+I), and dependent (+D). I believe that this GO term could be well modeled as a material role, having OntoClean meta-properties such as (−R, +I, +D), and it could be subsumed by the type called “molecular function,” resulting to more semantic clarity. In the BFO, role has been subsumed by dependent entity which is subsumed by continuant entity (Kumar and Smith, 2003). Placing role under “property” which isA DOLCE:Universal, rather than assuming role enduring self-identically through time as is in BFO (Kumar and Smith, 2003) seems to me as a better choice.
- In the Open Biomedical Ontologies (OBO) (http://obo.sourceforge.net), relations lack explicit formal definitions creating the possibility of confusions. Inadequacies could also be found in the use of relations such as is_A and part_Of (Smith et al., 2005; Burek et al., 2006). The distinction between function and their functioning in the GO has also been confusing, though a solution was attempted by the GO by appending the term “activity,” e.g., “galactokinase activity” (Krummenacker et al., 2009). Another problem which could arise from the use of multiple inheritances and is_A overloading is polysemy (Guarino, 1999). The problem of multiple inheritance in its conceptual hierarchies prevents it from logical reasoning applications. To understand one such inadequacy, let's take an example from the GO described graphically in Krummenacker et al. (2009). If galactokinase activity is made a subclass of carbohydrate kinase activity and phosphotransferase activity, then as per the rules of subsumption (Guarino, 1998), it would inherit the identity of both the super-classes. But, I believe this creates confusion, since the identity criteria of carbohydrate kinase activity would be different from the identity criteria of phosphotransferase activity, and any prediction based on such a hierarchy could lead to erroneous results. Though it may also appear as a semantic duplication in the ontology, the reasons why I feel it is important are: (1) lack of maintainability, (2) increased chances of confusion/inconsistency, (3) reduced search time efficiency, and (4) extra storage space. The formal logical modeling techniques in OntoClean method and top-level ontological distinctions between “universal” and “particular” in DOLCE, both having well founded formal semantics, could be used to understand better the underlying ontological structure and semantics of the classes and avoid polysemy.

## Conclusions and future directions

Biological ontologies are plagued by deficiencies in conceptual integration and inter-linkage (Beisswanger et al., 2007), and lacks sufficient concepts to represent functioning/actions/events (Schulz, 2006). The primary aim of this paper is to argue for the use of DOLCE (supported by OntoClean methods) as an upper level (or foundational) ontology, to describe general concepts shared by several biological domain ontologies, and to align them. As a semantic web agent may use several domain ontologies, aligning the domain ontologies becomes crucial to reduce semantic mismatch among services. Arguably, mathematical knowledge, comprising both symbolic notations and natural language, remains largely under-represented for semantic web agents. Though MathML and OpenMath have been developed to be used with Resource Description Framework (RDF), their success have been limited by the vocabulary provided by the ontology. As DOLCE (and OntoClean) have not been used so far as a foundational ontology for aligning many widely used biological domain ontologies, this discussion is intended as a motivation for a more detailed future research on it. As an example of how DOLCE could capture ontological categories underlying mathematical knowledge, the parthood relation in DOLCE could be used to represent: “a symbol is part_Of a formula.” Complementarity of foundational ontology and domain ontologies is believed to serve as a corrective to each others individual pitfalls. Detailed analysis of how DOLCE can satisfy all the requirements to represent mathematical knowledge is left as a future work.
